# The Impact of Oxidative Stress on Ribosomes: From Injury to Regulation

**DOI:** 10.3390/cells8111379

**Published:** 2019-11-02

**Authors:** Natalia Shcherbik, Dimitri G. Pestov

**Affiliations:** Department of Cell Biology and Neuroscience, Rowan University School of Osteopathic Medicine, Stratford, NJ 08084, USA

**Keywords:** ribosomal RNA (rRNA), ribosomal proteins, translation, reactive oxygen species, oxidative damage, iron homeostasis, Fenton reaction, stress response

## Abstract

The ribosome is a complex ribonucleoprotein-based molecular machine that orchestrates protein synthesis in the cell. Both ribosomal RNA and ribosomal proteins can be chemically modified by reactive oxygen species, which may alter the ribosome′s functions or cause a complete loss of functionality. The oxidative damage that ribosomes accumulate during their lifespan in a cell may lead to reduced or faulty translation and contribute to various pathologies. However, remarkably little is known about the biological consequences of oxidative damage to the ribosome. Here, we provide a concise summary of the known types of changes induced by reactive oxygen species in rRNA and ribosomal proteins and discuss the existing experimental evidence of how these modifications may affect ribosome dynamics and function. We emphasize the special role that redox-active transition metals, such as iron, play in ribosome homeostasis and stability. We also discuss the hypothesis that redox-mediated ribosome modifications may contribute to adaptive cellular responses to stress.

## 1. Introduction

Life in an oxygen-containing environment involves dealing with the omnipresent products of incomplete reduction of oxygen, known as reactive oxygen species (ROS) [1,2]. Depending on context and concentration, ROS can either play essential biochemical and signaling roles, or inflict damage on cellular components [3]. To minimize ROS-mediated damage, cells developed a variety of defensive enzymatic and nonenzymatic mechanisms that keep ROS levels within the optimal or tolerable range. When the load of ROS exceeds the capacity of the cellular antioxidant systems, a multitude of cellular functions may be affected, a state referred to as oxidative stress [4].

How oxidative stress affects the process of protein synthesis is a question of considerable interest for biomedical research. Excessive ROS generation is a recurrent molecular feature in the pathology and treatment of human disease, including neurodegeneration, cancer, and vascular disorders [5,6,7]. Many studies have shown that oxidative stress inhibits protein synthesis in cells [8,9]. However, a severe reduction of translation could be detrimental during stress as it is precisely the time when cells require new protein synthesis in order to repair damage and adapt to the new environment [10]. Recently developed technologies, such as ribosome profiling and quantitative proteomics, have revealed large-scale alterations in the translational landscape during oxidative stress, often preceding transcriptional changes [11,12]. Quick changes in translation may allow cells to more effectively react to adverse conditions, thereby reducing the extent of ROS-inflicted damage [13].

Due to the fact that the protein biosynthetic machinery is complex, it presents numerous control points for stress response regulation. However, complex machinery can malfunction when its critical parts are damaged. Studies using model organisms from every domain of life have uncovered ROS-induced chemical alterations in aminoacyl-tRNA-synthetases and protein factors involved in the initiation, elongation, and termination of translation [8,14,15,16,17,18,19]. Exposure to oxidants affects posttranslational modifications and the stability of tRNAs [20,21,22]. In some cases, ROS-mediated cleavages of mature tRNAs can result in the production of tRNA fragments [23,24,25] that either reprogram or inhibit translation [26,27,28,29]. Other studies demonstrated that damage to mRNAs by ROS may both stall translation and induce translational errors [30,31,32].

Among translation components, the impact of oxidative stress on the central player in this process, the ribosome, remains the least studied. Ribosomes are large ribonucleoprotein-based molecular machines composed of two subunits; the large subunits (LSU) in cytoplasmic ribosomes are referred to as 60S in eukaryotes and 50S in prokaryotes, while the small subunits (SSU) are called 40S and 30S, respectively. Each ribosomal subunit is built around intricately folded RNA molecules (rRNAs), which are bound by several dozen ribosomal proteins (r-proteins) [33]. In addition to cytoplasmic ribosomes, distinct types of ribosomes are present in mitochondria and plastids. As these energy-generating organelles produce large quantities of ROS, protecting their ribosomes against oxidative damage may require additional safeguard mechanisms, details of which are only beginning to come to light. In this review, we focus primarily on cytoplasmic ribosomes and first provide a concise summary of the known types of chemical changes in ribosomal components, induced by increased levels of ROS. We then discuss the impact of oxidative damage on ribosome function and outline areas for future exploration.

## 2. Oxidative Modification of rRNA

rRNA constitutes the structural and functional core of the ribosome. ROS can affect RNA in a multitude of ways [34,35,36,37], including chemical modification of the base and sugar moieties [38], generation of abasic sites [39], and strand breaks [40]. Guanine is the most easily oxidized nucleobase [41] and best studied in this respect. An oxidized form of guanine, 8-oxo-7,8-dihydroguanine (8-hydroxyguanine; 8-oxo-G), is a ubiquitous oxidative lesion, readily detectable in cellular nucleic acids [34,42]. When present in mRNA, 8-oxo-G interferes with decoding, possibly through the formation of a Hoogsteen pair with adenine when the base rotates around the N-glycosidic bond [43]. The altered base-pairing capacity of 8-oxo-G can also perturb RNA folding. A recent study revealed a variety of outcomes when 8-oxo-G was incorporated into model RNA substrates, from stabilization of existing structural motifs to their destabilization and rearrangements into new structures [44]. Since ribosome activities in translation depend on many precisely tuned conformational changes and movements within its rRNA framework [45], oxidation of the bases that are critical for maintaining the correct rRNA structure may impair ribosome functions. It seems therefore likely that the need to withstand a certain degree of oxidation was one of the forces in shaping the evolution of the ribosome. A striking disappearance of many guanines from mitochondrial rRNA [46] and reduced overall RNA content [47] may be two such evolutionary adaptations that “hardened” mitoribosomes against damage from the abundant ROS generated inside mitochondria.

The extent of cellular RNA oxidation was reported to exceed that of DNA when cells were directly treated with H_2_O_2_ [40,48] or subjected to conditions that induce secondary oxidative damage, such as UVA irradiation [49] and ammonia overload [50]. In one study [40], exposure of *E. coli* cells to H_2_O_2_ was shown to induce a dosage-dependent increase of 8-oxo-G in all tested RNA types, including rRNA. By comparing 8-oxo-G levels after in vitro H_2_O_2_ treatment of RNAs in their native and denatured forms, the same study concluded that folding of rRNA did not protect it against oxidative damage [40]. Similar conclusions were made by Willi and colleagues, who used an 8-oxo-G antibody to immunoprecipitate oxidized RNA from H_2_O_2_-treated *E. coli* cells [51].

A large body of observations points to the association of oxidized RNA with disease [52,53,54]. High 8-oxo-G content in cytoplasmic and nucleolar RNA was found in vulnerable neurons in Alzheimer′s disease (AD) [55], Parkinson’s disease [56], and neurons of the hippocampal region and temporal neocortex in patients with Lewy body dementia [57]. Elevated levels of 8-oxo-G in RNA were also detected in neuropsychiatric disorders, including schizophrenia, depressive, and bipolar disorders [58]. Furthermore, RNA extracted from atherosclerotic plaques was heavily modified with 8-oxo-G [59]. Although links between 8-oxo-G and human pathologies have been based largely on the analysis of total RNA, rRNA is the most abundant cellular RNA type, which typically accounts for about 80% of the total RNA [60], from which one can reasonably assume that oxidative damage to ribosomes in all these pathological conditions must be widespread. Studies that focused specifically on rRNA confirmed it to be a major target of the 8-oxo-G modification in patients with AD and mild cognitive impairment, a condition that often precedes AD [61,62].

## 3. Reversible Oxidation of Cysteine and Methionine in r-Proteins

The cyclic oxidation and reduction of cysteine (Cys) and methionine (Met) residues in a protein represents an important mechanism in the regulation of protein functions. The sulfur atom present in the fully reduced free thiol (-SH) side chain of Cys can be oxidized to the disulfide bond (Cys-S-S-Cys), sulfenic (-SOH), sulfinic (SO_2_H), and sulfonic acids (-SO_3_H), thereby making Cys an exquisite redox stress sensor [63]. Analysis of the data generated by semiquantitative mass spectrometry coupled with thiol trapping suggests that some r-proteins may be oxidized during normal growth in yeast cells [64]. Similar results were obtained in the human cell line HT-29 [65]. R-proteins represented a prominent cluster in the quantitative whole-cell redoxome analysis using oxidative isotope-coded affinity tags (OxICAT) in different organisms including *E. coli* [66], *S. cerevisiae* [9,67], *C. elegans* [68], and *D. melanogaster* [69]. In all cases, OxICAT identified r-proteins from both subunits, suggesting that oxidation of Cys residues may influence various stages and features of translation. Based on yeast studies, Topf and colleagues further proposed that r-proteins can act as redox sensors, mediating the attenuation of protein synthesis in response to oxidative stress [9]. Exactly which r-proteins might affect the translation inhibition remains to be determined. It is also currently unclear whether attenuation of protein synthesis represents the main outcome of thiol oxidation in r-proteins. Considering that OxICAT identified a number of r-proteins in which oxidative stress affects redox state, it is tempting to speculate that different patterns of oxidation in r-proteins could lead to variable translational remodeling as a means to adapt to oxidative stress, although more studies will be needed to test this hypothesis.

Similar to Cys, Met residues can be readily oxidized, leading to the formation of methionine sulfoxide [70]. Little is known about the potential contribution of oxidized Met residues to changes in ribosome function. Two independent studies published in 1978 reported oxidation of Met residues of the L7/L12 r-protein in bacterial ribosomes [71,72]. L7 and L12 are identical, except that L7 is acetylated at the N terminus; L7/L12 are normally present on *E. coli* 50S subunits as two dimers that constitute the L7/L12 stalk, important for interactions with translation factors [73,74]. It was found that oxidation of the Met residues in L7/L12 abolished the dimer formation, affecting stalk structure and altering the ability of the bacterial ribosome to interact with the elongation factor EF-G [71,72].

## 4. Oxidative Damage to r-Proteins

The effects of ROS on proteins are mediated to a large extent through initial modifications of cysteine, methionine, tyrosine, tryptophan, and histidine residues, which in turn may give rise to a complex set of secondary reactions that are damaging for the proteins and other biological molecules with which they interact [75,76,77]. One important type of modification is carbonylation, mostly affecting arginine, lysine, proline, threonine, and a few other amino acid side chains [78]. Whereas cells maintain enzymatic systems that can repair oxidized Cys and Met residues [79], carbonylation is not repairable and may irreversibly damage and/or destabilize proteins. Several excellent reviews discuss carbonylation mechanisms in detail [77,80,81,82].

Carbonylation presents technical challenges for detection because it generates a wide variety of adducts [83]. For example, oxidation of tryptophan alone results in the formation of seven potential carbonylated products [84,85,86]. Nevertheless, techniques have been developed to reliably identify carbonylated proteins, modification sites, and types of carbonylation by stabilizing carbonyl moieties followed by chromatographic enrichment and proteomics-based analysis [81,87,88]. A recent high-throughput screen to examine carbonyl-modified proteins detected components of the translation machinery, including several amino-acyl tRNA synthetases and two r-proteins (Rpl32 and Rpl35) in HeLa cells treated with low doses of H_2_O_2_ (0.05 mM–0.5 mM) [89]. In another study, Mirzaei and Regnier found that a major group of proteins carbonylated in *S. cerevisiae* in response to a high-dose (5 mM) H_2_O_2_ treatment is comprised of ribosomal and ribosome-associated proteins [90,91].

Among the reactive derivatives produced by oxidation of cellular components, aldehydes such as malonaldidehyde (MDA) and 4-hydroxy-2-nonenal (HNE) operate as agents of secondary protein carbonylation. In cells, both of these aldehydes originate from the ROS-induced peroxidation of fatty acids and are commonly used as oxidative stress markers [80]. In human colorectal carcinoma RKO cells, 18 translation-related HNE-modified proteins, including six r-proteins, were significantly increased after treatment with different doses of HNE [92]. How modification with this product of lipid peroxidation may affect ribosome function remains unknown.

In addition to carbonylation, proteomic studies provided evidence of widespread crosslinking between r-proteins and the ribosome′s rRNA backbone in response to acute oxidative stress. In yeast cells treated with high doses of H_2_O_2_, 37 r-proteins were reported to form cross-links with RNA [91]. The functional impact of the rRNA-protein crosslinking is yet to be determined.

## 5. Ubiquitination of r-Proteins Following Oxidative Stress

In addition to attacking ribosome components, ROS may alter activities of ribosome-modifying enzymes and thus affect the ribosome indirectly. The modification of eukaryotic ribosomes with the 76-amino acid polypeptide ubiquitin (Ub) is one such example. Ubiquitination of r-proteins on ribosomes made nonfunctional by a mutation in rRNA was first observed a decade ago [93], and later shown to occur on ribosomes isolated from cells treated with H_2_O_2_ [94].

Ub moieties can be covalently attached to Lys residues present either on the target protein or on another Ub polypeptide. Given that Ub contains seven Lys residues, different types of poly-Ub chains can be assembled, including those linked through Lys48 (K48-Ub chains) or through Lys63 (K63-Ub chains). It is generally accepted that the K48-Ub chains primarily label proteins for degradation by proteasomes, while the K63-Ub chains play regulatory roles [95]. By applying a new mass spectrometry-based technique called Ub-DiGGer to detect specific Ub linkages, Silva and colleagues have recently shown that ribosomes are prominent targets of polyubiquitination with K63-Ub chains [96,97]. Modification with K63-Ub chains was detected in r-proteins in yeast and mammalian cells grown under normal conditions and found to increase during oxidative stress. The K63-Ub chains decorated r-proteins located on the solvent-exposed surfaces of the ribosome and were particularly abundant around the head of the 40S subunit. The yeasts Rad6 and Bre1 were identified as the E2/E3 enzymes responsible for the ubiquitination of r-proteins with K63-Ub chains, while deubiquitinase Ubp2 was implicated in removing the K63-Ub chains from the ribosome. On the mechanistic side, the control of the Rad6/Bre1-Ubp2 circuit is accomplished through the oxidation of a catalytically active Cys residue in Ubp2; this oxidation inactivates the enzyme, shifting the balance towards the ubiquitinated ribosomes. The same group later identified r-proteins as a major group of oxidized cellular targets that undergo K48-Ub chain polyubiquitination and subsequent degradation [98]. The attachment of K48-Ub chains was found to occur in two major stages: as an immediate response to oxidative stress and during the recovery period. Together, these findings illustrate the complexity of r-protein ubiquitination and hint at a potentially important role of this type of ribosome modification as a regulatory mechanism in the changing redox environment.

## 6. Role of Metals in the Effects of Oxidants on the Ribosome

Although readily diffusible, H_2_O_2_ reacts poorly with most biological macromolecules [99]. In DNA studies, it has been well established that mutagenic properties of H_2_O_2_ depend largely on its reactions with redox-active transition metals [1]. The best documented example involves ferrous iron (Fe^2+^), which can damage biomolecules through the generation of the highly reactive hydroxyl radical (OH^•^) in the Fenton reaction:

L-Fe^2+^ + H_2_O_2_ → L-Fe^3+^ + OH^•^ + OH¯.

When the ligand (L) represents a nucleic acid or a protein, metal binding often occurs in a sequence and structure-dependent manner. This creates active centers for localized generation of OH^•^, resulting in site-specific damage [100,101]. Complexes of higher oxidation states of iron can damage macromolecules through additional mechanisms that are dependent on the nature of the ligand and are not always easy to precisely define [99].

In our recent studies, we have found that intracellular iron is responsible for site-specific cleavages in yeast rRNA that occur in ribosomes when cells experience oxidative stress [102,103]. One prominent cleavage in the expansion segment 7 (ES7L) of 25S rRNA was detectable by northern analysis within one minute of treating yeast cells with ROS inducers and could also be reproduced by redox cycling of iron bound to purified ribosomes in vitro [103]. Iron binding to ribosomes in cells appears to play roles that, depending on context, could be either regulatory or destructive. Iron-mediated cleavage in ES7L occurs in wild-type cells during mild redox perturbations that make cells more resistant to subsequent oxidative challenges [102], suggesting a role in the adaptive response to stress. Mutations that lead to perturbations in iron homeostasis, by contrast, provoke excessive rRNA fragmentation and loss of cell viability after mild oxidant challenges, from which wild-type cells fully recover [103].

When tested in anoxic conditions, Fe^2+^ ions can functionally substitute Mg^2+^ in rRNA folding and cell-free translation, owing to the close geometry of the RNA-Mg^2+^ and RNA-Fe^2+^ complexes and possibly reflecting the ribosome′s origins in an iron-rich and oxygen-poor prebiotic environment [104,105]. Given iron′s potential to promote free-radical formation, it is not surprising that its metabolism in aerobic organisms is tightly regulated [106]. Notably, in vitro reactions using high concentrations of Fe^2+^ and H_2_O_2_, similar to those used for hydroxyl radical footprinting [107], produce massive degradation of rRNA [108]. It is generally accepted that most intracellular iron is chelated but can be exchanged between its biological ligands [109]. Thus, under normal circumstances when intracellular iron is properly controlled, iron is more likely to promote limited, site-specific modifications in ribosomes upon their encounter with ROS. It is noteworthy that aside from RNA strand cleavages, iron can potentiate many other types of covalent RNA and protein modifications induced by ROS [101]. In a way, iron binding can make the ribosome more “redox-aware”.

Binding of other metals can also induce chemical changes in biological macromolecules, either through metal ions directly reacting with the bound molecules or by facilitating localized generation of ROS. For example, amino acid residues next to various metal-binding sites in proteins have been shown to be highly prone to metal-catalyzed oxidation (MCO) [77], which could represent another mechanism for metal-dependent ROS effects on the ribosome. Binding of Pb^2+^ to purified *E. coli* ribosomes was shown to generate site-specific cleavages in rRNA, which were counteracted by divalent metal ions competing for binding to the same sites [110]. As an illustration of the contribution of environmental metals to ribosome damage, studies in mussels exposed to Cu^2+^, Cd^2+^ and Hg^2+^ found increased levels of 8-oxo-G and strand breaks in rRNA [111]. It will be important to determine to what extent metals may be responsible for potentiating ribosome damage in other situations, including human disease. As discussed above, accumulation of oxidative damage in rRNA is observed in brain tissues in a number of pathological states associated with disruptions in proteostasis [53,55,61,62]. Likewise, abnormal iron homeostasis and its altered cellular distribution is a well-documented feature in brain pathologies, including neurodegenerative conditions such as AD and Parkinson′s diseases, as well as traumatic brain injury [112,113,114]. Copper is another transition metal capable of promoting redox damage to nucleic acids and proteins [100], with known links to neurodegeneration [115].

## 7. How Oxidative Modifications of the Ribosome May Affect Cellular Functions

Although a variety of modifications in ribosomal components caused by oxidative stress have been identified to date (Table 1), few studies have attempted to address the biological role of these modifications. Subjecting ribosomes to an intense oxidant treatment was shown to reduce incorporation of labeled Phe into products of cell-free translation reactions driven by poly(U) [108]. Oxidative damage to specific nucleobases or specific amino acid residues in r-proteins is probably more physiologically relevant, but also more difficult to address experimentally. A recent study by the Polacek group [51] stands out for its elegant use of an atomic mutagenesis approach to reconstitute *T. aquaticus* 50S subunits with a single oxidized base at several functionally important positions around the ribosome′s peptidyl transferase center. Remarkably, while some of these single-base substitutions were found to inhibit translation of model mRNA substrates in vitro, others had no measurable effect or even increased the yield of the translated product. These varied outcomes highlight the complexity of oxidant effects on ribosome functions in translation. The situation is likely even more complex in vivo, where ribosomes have to synthesize much more challenging polypeptides and correctly process numerous regulatory signals.

As already mentioned, aside from promoting direct oxidation of ribosomal components, oxidative stress may also influence activities of ribosome-modifying enzymes. Given the dearth of mechanistic studies, for the most part we can currently only speculate about the functional significance of individual modifications. One plausible scenario is that an altered behavior of the modified ribosomes might contribute to the rapid translational shifts that occur after cell exposure to oxidants [12]. Other modifications might mark individual ribosomal subunits for a mechanical action by cellular enzymes, sequestration, or degradation. Indeed, recent studies identified yeast Rps3 (uS3) as a ubiquitination substrate that controls the release of the 40S subunit from translational stalls, followed by the subunit′s decay [116,117]. Ub modifications of the ribosome were also observed in yeast during starvation as part of ribophagy, a specialized form of autophagy that removes excessive ribosomes to the vacuole [118], and during the unfolded protein response to proteotoxic stress [119], known to perturb redox balance in cells [120,121].

While changes in r-protein ubiquitination manifest prominently after oxidant treatments, this likely represents only the tip of the iceberg. A recently performed affinity purification of mammalian ribosomes identified several hundred ribosome-associated proteins including RNA- and protein-modifying enzymes, as well as proteins involved in redox regulation [122]. Another group of potentially interesting redox effectors are Fe(II)/2-oxoglutarate (2OG)-dependent oxygenases [123], shown to catalyze hydroxylation of Arg, Pro, and His residues in r-proteins in both prokaryotes and eukaryotes [124,125,126,127]. One 2OG-dependent oxygenase, RlhA, is responsible for hydroxylation of the cytosine residue C2501 in the peptidyl transferase center of the *E. coli* LSU [128] and hypothesized to play a role in environmental stress responses [129]. 2OG-dependent oxygenases depend on the presence of reduced iron in their active center [130] and might potentially modulate ribosome functions in a redox- and metal-dependent manner.

The specificity of ribosome modifications observed under mild oxidative stress, such as site-specific rRNA cleavages [102] or Ub modifications of specific r-proteins [96], is consistent with the idea that some of the oxidative stress-driven ribosome modifications may play regulatory roles. More studies are clearly needed to determine how redox-induced modifications in ribosomes might help cells to cope with various stress-inducing conditions.

Obviously, not all changes in ribosomes caused by ROS occur in the context of adaptive cellular responses. Fragmentation of rRNA has been observed in mammalian and yeast studies as part of programmed cell death [131,132]. Whether the destruction of the cell’s ribosomes helps to execute the apoptotic program or simply represents an end result of the nuclease release and/or increased ROS production during this process [133] remains unknown. The cause and effect relationships also remain insufficiently well understood in various cases of increased rRNA oxidation associated with organ and tissue pathology discussed above. Another series of intriguing observations links accumulation of oxidative lesions in RNA to age-related disease [134]. Among unicellular organisms, an increased level of protein carbonylation in ribosomal particles has been observed in the nonculturable population of cells from a stationary-phase *E. coli* culture [135]. The idea that the competence of the protein-synthetic apparatus may be one of the factors in the aging process, as proposed in the early days of molecular biology by Orgel [136], has been difficult to prove, but as more information about ribosome damage becomes available, this point warrants a thorough reinvestigation.

## 8. Mechanisms for Dealing with Ribosome Damage: Reduce, Repair, Recycle

Cells have evolved enzymes to eliminate 8-oxo-GTP from the nucleotide pool as a way to reduce its incorporation into nucleic acids [137,138,139,140]. Thus, newly synthesized ribosomes should be relatively free of oxidative lesions in their rRNA, but they may accumulate oxidative damage during their lifetime in the cell. A substantial amount of oxidation was detected in rRNA isolated from *E. coli* cells, even if the cells were not subjected to any special oxidant treatment [51]. Interestingly, *E. coli* cells under normal growth conditions were found to contain lower levels of 8-oxo-G in rRNA than the rest of cellular RNA [40]. Theoretically, this could be due to either efficient elimination of damaged ribosomes from cells or the presence of some kind of repair mechanisms specific for ribosomes. Repair systems are essential for neutralizing the oxidative damage in DNA molecules incurred in the aerobic environment [1,141]. It is generally assumed that damaged RNA is degraded rather than repaired, a notion well supported by studies of mRNA [34,36,142]. Unlike mRNAs, which can be relatively easily remade, ribosomes are large and complex molecular machines that are expensive for the cell to build; destroying the entire ribosome seems a wasteful strategy if damage is repairable. In fact, replacement of some damaged r-proteins in fully assembled ribosomes has been already demonstrated [143]. Repair of oxidized Cys and Met residues in r-proteins should, in principle, also be possible through the activities of oxidoreductases such as thioredoxins, glutaredoxins, and methionine sulfoxide reductases [79]. To date, the most compelling case for repair of environmental damage in RNA is provided by oxidative demethylases, enzymes shown to reverse alkylation damage in both DNA and RNA [144]. It remains to be seen whether this and other types of damage to rRNA molecules, including oxidative damage, can be repaired in cells.

A number of nucleases have been implicated in the decay of rRNA in cytoplasmic ribosomes [145,146,147,148]. To effectively participate in oxidative damage control, these enzymes must either recognize oxidative lesions themselves or be guided to the damaged ribosomes by cofactors. A few examples of selectivity towards oxidative lesions in RNA have been described for human proteins [149,150], although it is unknown whether these proteins might contribute to decay of damaged rRNA. Lack of bacterial polynucleotide phosphorylase (PNPase), which binds to oxidized RNA, was shown to cause higher accumulation of 8-oxo-G and hypersensitivity of cells to H_2_O_2_ [151,152]. A similar behavior was reported for the human mitochondrial PNPase, although the precise mechanism by which PNPase promotes the selective reduction of oxidized RNA in the cell requires further investigation [153]. Studies in algae suggested a role for RBCL, a subunit of the Rubisco protein, in the management of oxidized RNA within chloroplasts [154]. Mechanisms that counteract oxidative damage may also operate during ribosome assembly steps. The apurinic/apyrimidinic endonuclease APE1, shown to cleave abasic sites within RNA in vitro [155], was found to accumulate in the nucleolus in interphase HeLa cells, making this protein well positioned to serve as a quality control factor in ribosome biosynthesis [156].

The complex folding of rRNA and its association with ribosomal proteins makes ribosomes rather difficult substrates for nucleases. How the large ribosomal particles are dismantled for the effective digestion of rRNA is not well understood. Studies demonstrating the role of the ubiquitin-proteasome system in eukaryotic ribosome degradation [93,94,117] point to the possible contribution of components of this system to the disassembly of ribosomal particles. Interestingly, inhibition of the proteasome in primary neuron and astrocyte cultures was found to strongly increase 8-oxo-G content in RNA, accompanied by diminished 18S and 28S rRNA levels and the appearance of cross-linked RNA species [157]. Autophagy is another general mechanism that eukaryotic cells utilize to eliminate excessive ribosomes [158]. Starvation conditions have been shown to greatly increase autophagic delivery of ribosomes to the lysosome, a process termed ribophagy [118,159,160]. The idea that lysosome-targeting ribophagy mechanisms [118,161] may also operate on damaged ribosomes is attractive, but still awaits direct experimental confirmation. Using ribosomal protein fusions with the pH-sensitive reporter Keima, An and Harper recently observed a modestly increased ribophagic flux after treatment of HEK293 cells with H_2_O_2_ [159]. Future studies will be needed to elucidate details of ribosome degradation mechanisms and determine the roles of the cytosolic and lysosome machineries in removing oxidatively damaged ribosomes from the translating pool.

## 9. Conclusions and Perspectives

**Figure 1:**
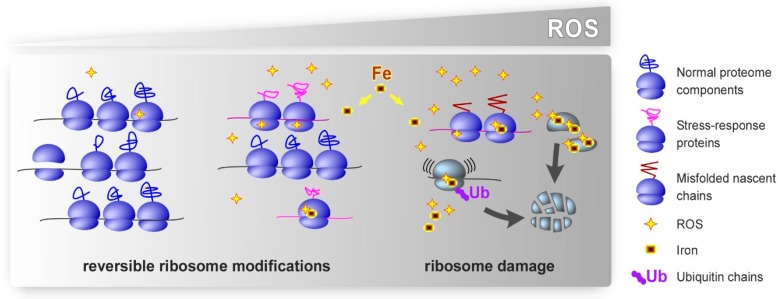
Putative effects of oxidative stress on ribosomes. Cellular oxidative defenses neutralize excess ROS under basal conditions, which permits normal translation. We propose that low-level oxidative stress leads to largely reversible modifications in rRNA and r-proteins. These modifications could potentially promote selective translation of stress-response proteins and facilitate adaptive cellular responses. Strong or chronic oxidative stress inflicts excessive damage to ribosomes. Damaged ribosomes may be prone to making errors, become stalled during translation, or synthesize polypeptides that fail to fold correctly. In addition to ROS, intracellular iron contributes to the effects of oxidants by binding to specific sites on ribosomes, where it promotes localized redox reactions. Ubiquitination of r-proteins, one function of which appears to target dysfunctional ribosomes for degradation, has emerged as a prominent type of the secondary modifications of ribosomes under oxidative stress conditions.

In contrast to the progress achieved in deciphering normal ribosome functions in translation, our understanding of the oxidative damage to ribosomes and its impact on cell physiology is still very limited. It is important to keep in mind that oxidative stress comes in many “shades of grey”, with variations in the intensity, duration, types and location of the generated ROS and their primary cellular targets [2]. Thus, it is likely that a spectrum of oxidative modifications of the ribosome exists in cells. One intriguing possibility, which still needs experimental validation, is that localized redox modifications of rRNA and r-proteins, occurring when ROS levels in a cell increase, might confer distinctive properties to some ribosomes, thereby facilitating adaptive processes such as translation of stress-response genes (Figure 1). In effect, the total cellular ribosomal pool could separate into functionally nonidentical subpopulations under stress, each one performing their duties differently from the others. Considering experimental challenges in the way of rigorously proving ribosome specialization [162,163], we expect that establishing the existence of such ROS-induced ribosome heterogeneity would similarly require a lot of supporting biochemical work. Severe oxidative stress is likely to result in widespread ribosome damage (Figure 1). As a result of the ribosome dysfunction, newly translated proteins may be inactive, interfere with normal cellular processes through dominant-negative mechanisms or promote pathological aggregate formation, all of which could have broad implications for human disease.

Based on the information available to us today, some of the challenging questions to be addressed in the future studies include the following:

Which ribosomal parts can undergo oxidation without losing functionality and which ones are easily damaged by oxidants?

How do ROS-induced ribosome modifications affect translation and to what extent do these modifications help cells to fight oxidative stress?

What are the consequences of ribosome damage for cell growth, survival, and tissue functions? How much ribosome damage can different cell types tolerate?

Is there a special degradation pathway(s) for ribosomes made dysfunctional by oxidative damage?

Another challenge will be to understand to what extent ROS-induced alterations in ribosome functions act as a driving force in disease etiology and progression, as well as the full diagnostic value of these alterations as a biomarker. Only a handful of studies to date have made an attempt to address these issues, and the presented evidence remains mostly circumstantial. It is clear that many interesting discoveries still lie ahead.

## Figures and Tables

**Table 1:** Ribosomal RNA and protein modifications induced by oxidative stress.

Type of Modification	Target	Organism	Reference
Guanine base	rRNA	*Escherichia coli*	[40,51]
oxidation		*Mytilus galloprovincialis*	[111]
		*Rattus norvegicus*	[50]
		*Homo sapiens*	[61,62,108]
Strand scission	rRNA	*Escherichia coli*	[40]
		*Saccharomyces cerevisiae*	[102,103,132]
		*Homo sapiens*	[131]
rRNA-protein cross-links	rRNA, r-proteins	*Saccharomyces cerevisiae*	[91]
Met oxidation	r-proteins (LSU)	*Escherichia coli*	[71,72]
Cys oxidation	r-proteins (LSU + SSU)	*Escherichia coli*	[66]
		*Saccharomyces cerevisiae*	[9,64,67]
		*Caenorhabditis elegans*	[68]
		*Drosophila melanogaster*	[69]
		*Homo sapiens (HT-29 cells)*	[65]
Carbonylation	r-proteins (LSU + SSU)	*Saccharomyces cerevisiae*	[90,91]
	r-proteins (LSU)	*Homo sapiens (HeLa cells)*	[89]
Adduct formation	r-proteins (LSU + SSU)	*Homo sapiens (RKO cells)*	[92]
Ubiquitination	r-proteins (LSU + SSU)	*Saccharomyces cerevisiae*	[94,118]
	r-proteins (SSU)	*Saccharomyces cerevisiae*	[116,117]
		*Homo sapiens (HCT116 cells)*	[119]
Ubiquitination	r-proteins (LSU + SSU)	*Saccharomyces cerevisiae*	[96,97]
(K63-Ub chains)		*Mus musculus (HT22 cells* *)*	[97]
Ubiquitination (K48-Ub chains)	r-proteins (LSU + SSU)	*Saccharomyces cerevisiae*	[98]
